# Effect of struvite (*Crystal Green*) fertilization on soil element content determined by different methods under soybean cultivation

**DOI:** 10.1038/s41598-023-39753-8

**Published:** 2023-08-05

**Authors:** Anna Jama-Rodzeńska, Bernard Gałka, Anna Szuba-Trznadel, Anita Jandy, Joanna A. Kamińska

**Affiliations:** 1 Institute of Agroecology and Plant Production, Faculty of Life Sciences and Technology, Wrocław University of Environmental and Life Sciences, 50-363 Wroclaw, Poland; 2 Institute of Soil Science, Plant Nutrition and Environmental Protection, Faculty of Life Sciences and Technology, Wrocław University of Environmental and Life Sciences, 50-363 Wroclaw, Poland; 3 Department of Animal Nutrition and Feed Science, Faculty of Biology and Animal Science, Wroclaw University of Environmental and Life Sciences, 51-630 Wroclaw, Poland; 4 Center for Environmental Quality Analysis, Institute of Soil Science, Plant Nutrition and Environmental Protection, Faculty of Life Sciences and Technology, Wrocław University of Environmental and Life Sciences, 50-363 Wroclaw, Poland; 5 Department of Applied Mathematics, Faculty of Environmental Engineering and Geodesy, Wrocław University of Environmental and Life Sciences, 50-363 Wroclaw, Poland

**Keywords:** Agroecology, Climate-change ecology

## Abstract

Struvite is regarded as a promising phosphorus fertilizer alternative to mineral fertilizers; however before fertilizing, soil tests should be undertaken to determine fertilizer recommendations. In May 2022, soil was sampled from a pot experiment with the application of phosphorus set up at the Wroclaw University and Environmental and Life Sciences. Chemical analysis of the soil included total and available phosphorus, potassium, magnesium determined by the Egner–Riehm, Mehlich 3 and Yanai methods. The purpose of the article is to compare soil element extraction by three different methods under struvite fertilization and its use in soybean cultivation. The application of these methods indicated an unambiguous increase in soil Mg content after struvite application. Broadcast soybean fertilization affected the phosphorus content of the soil. The results of the study indicated that different extraction methods presented different contents of P from soil. The content of available phosphorus was circa 122–156 mg kg^−1^ dm, 35.4–67.5 mg kg^−1^ dm and 100–159 mg kg^−1^ dm according to the Mehlich, Yanai and Egner–Riehm methods, respectively. A positive correlation was found between the content of Mg and K in soil determined by the Mehlich 3 and Yanai methods, which may suggest that the Yanai method could be introduced into standard soil chemical analysis in Poland. Such a correlation was not found for phosphorus, which is a difficult element to determine due to the multitude of factors affecting its availability.

## Introduction

Phosphorus is an important element enhancing the nutrient richness and fertility of soils. It is also a pivotal component, responsible for the proper functioning of plants and leading to high quantity and good quality yields. The actual extent of commercially viable global phosphate rock reserves has remained a subject of considerable uncertainty in recent years^1^. It is estimated that at the current rate of use, the global phosphorus (P) reserve is sufficient for 600–1000 years^2,3^. P rock reserves are being depleted, thus threatening long-term global food security. Thus, alternatives for P are being investigated.

A promising possibility is struvite (MgNH_4_PO_4_6H_2_O), which can be recovered from sewage sludge. Struvite recovery has several additional advantages not only in agriculture sector but also in wastewater treatment plants^4–6^, as struvite is regarded as an alternative source of elements such as phosphorus, nitrogen and magnesium for agricultural purposes^7–9^. Struvite has a theoretical P content close to that of phosphate rock (12.6% dry weight [DW]) and has been shown to be an effective P fertilizer especially in acidic soils and is considered a slow-release fertilizer that can reduce P losses to the environment^10,11^. Struvite also contains some heavy metals, as wastewater contains a significant amount of such elements; however, these occur within acceptable limits, as has been proved in our own research^12^. Experimental data from various plant species proves that struvite fertilization results in similar plant yields to those achieved with mineral fertilization^7,8,12–15^.

Among nutrients, phosphorus in soil is one of the most difficult to analyse because of its various forms, such as P dissolved in soil solution; P absorbed in clay minerals, Fe and Al (hydr-)oxides; P in primary minerals organic P; and microbial P pools^16^. Phosphorus content is usually analysed with soil tests that have been developed over the last six decades^17–19^. Interpretation of phosphorus tests in soil is subject to considerable uncertainty. Soil phosphorus tests attempt to present plant uptake by extracting all or a proportional amount of this element available to plants. Examining the phosphorus content of the soil will help determine the appropriate phosphorus dose^20^. Numerous tests are used worldwide to determine soil P content, with more than 13 phosphorus tests developed for agronomic recommendations in America. Test selection usually depends on local soil conditions (e.g. some tests are better prepared to high or low pH conditions than others), although historical and institutional factors mainly influence test selection in different areas. Originally, all soil tests were analysed colorimetrically, such as the molybdenum blue method devised by Murphy and Riley^21^. After the implementation of inductively coupled plasma (ICP) spectrometry, new soil tests were developed in the 1970s and 1980s, allowing simultaneous measurement of many elements from a single soil extract^16^. In Poland, for a number of years, the Egner–Riehm method has been used to determine the abundance of soil in plant-available phosphorus and potassium^22^. It involves extracting phosphorus compounds from the soil using hydrochloric acid-acidified calcium lactate (CH_3_-CHOH-COO)_2_Ca). The solution used for extraction is 0.04 N to calcium lactate and 0.02 N to hydrochloric acid^22^.This method however, often proves insufficiently precise for the accurate determination of optimal plant doses of phosphorus to ensure high efficiency of fertilization and maintain at least average abundance of soil in plant-available phosphorus. Therefore, other, more complicated methods allowing the determination of the abundance of soil in different fractions of phosphorus should be used.

Mehlich 3 is a pivotal part of fertilizer recommendations for phosphorus (P), potassium (K), calcium (Ca), magnesium (Mg) and some trace elements to achieve optimum yield; however, it is not so popular in Poland in chemical and agricultural stations^23^. This method is mainly used in the Czech Republic and in major parts of Canada and the USA^24^. The intended purpose of this test is to isolate portions of several different P pools that are correlated with the amount of phosphorus that is available to plants during the growing season^23^. The rationale for implementing the Mehlich 3 method for agrochemical testing in Poland is that it provides a relatively simple determination of potassium, calcium, sodium, magnesium, iron, manganese, copper, zinc, boron, sulfur and especially phosphorus in a single extracted soil extract. The determination of phosphorus content by the Mehlich 3 method is mainly influenced by soil factors such as soil type, clay content, mineralogy and pH. The universal extraction solution (pH 2.5 ± 0.01) is designed for the determination of soil samples with acidic and neutral pH which constitute the majority of soils used agriculturally in Poland. The disadvantages of Mehlich 3 are as follows: NO_3_^−^N cannot be evaluated; the F− ion in the extractor can dissolve K from glass bottles; and EDTA in the extractor precipitates after prolonged storage^25^. Another method that can be used instead is the Yanai method, where one single extracting solution is used to simultaneously to extract NO_3_^−^N, available P and B, exchangeable K, Ca, and Mg, easily reducible Mn, and HCl-soluble Zn and Cu^25,26^.To minimize analysis time and laboratory costs, research is focusing on developing multi-nutrient methods that can be used to analyse macro and micro nutrients from the same extract. A modified multi-element method was proposed by Yanai^25,26^ where the composition of the extraction solution is as follows: 0.2 M CH_3_COOH, 0.25 M NH_4_Cl, 0.005 M citric acid and 0.05 M HCl. In addition, it can be used to determine the nitrate content of the soil due to the fact that there are no nitrate ions in the composition of the solution^26^.

In the working hypothesis, we assumed that the P as well as Mg content of struvite-applied soil would increase in the soil determined by all methods, and that the Yanai method would be as promising as Mehlich 3 in analysing P and K in soil.

The objectives of the present study were to: (1) understand the effect of struvite fertilization on P, K, Mg content in soil under soybean fertilization; (2) compare three different laboratory methods for the analysis of element content in soil obtained by a pot experiment; and (3) select the most appropriate method for analysing element content.

## Results

### Effect of struvite placement on seed yield and uptake of selected macroelements

There was no significant effect of the studied factors on soybean yield, i.e. K, Mg and P intake. Indeed, the most phosphorus was found in soybean under struvite fertilization (Table 1).

**Table 1:** Effect of fertilization and struvite placement on seed yield and uptake of macroelements (P, K, Mg).

Method of fertilizer placement	Phosphorus fertilizer	Yield	Mg (g kg^−1^)	K (g kg^−1^)	P (g kg^−1^)	Mg uptake (kg ha^−1^)	K uptake (kg ha^−1^)	P uptake (kg ha^−1^)
Band		3.50	2.72	17.16	8.04	9.60	60.13	28.20
Broadcast		3.87	2.69	14.56	7.94	10.49	56.76	30.95
p value		ns	ns	0.05*	ns	ns	ns	ns
	Control	3.43	2.77	16.28	7.71	9.53	56.02	26.55
	Superphosphate	3.69	2.61	15.61	7.92	9.78	57.75	29.35
	Struvite	3.93	2.74	15.56	8.29	10.82	60.88	32.55
p value		ns	ns	ns	0.05*	ns	ns	ns
A × B		ns	ns	ns	ns	ns	ns	ns

*Statistically significant α = 0.05; *ns* not significant statistically.

### Effect of fertilization and struvite placement on total and available forms of K, P and Mg in the soil

The effects of the method of fertilizer placement and various phosphorus fertilizers on K, P and Mg content in soil under soybean cultivation are presented in Table 2. The greatest content of K, P, Mg and pH was determined under band fertilization, while that for salinity was noted under broadcast fertilization (Table 2). Under struvite fertilization, the highest contents of the examined elements were seen under struvite fertilization. Struvite fertilization caused an increase in the examined elements compared to the control in the case of Mg, and the control and superphosphate in the cases of both K and P. Phosphorus fertilization decreased salinity in the soil. With regards to interaction between the examined factors, fertilization band caused a decrease in K content in the soil and an increase in the case of broadcast fertilization. The highest content of P was noted under struvite band fertilization. Interaction between the examined factors caused a decrease in soil salinity. Magnesium content also increased after struvite fertilization: by 17% compared to the control (all differences statistically not significant).

**Table 2:** Content of total forms of K, P and Mg (g kg^−1^) determined after microwave mineralization (*p*-value for the *t*-test with control group).

Method of fertilizer placement	Phosphorus fertilizer	K	P	Mg	pH	Salinity
Band		1.682	0.366	0.873	5.84	37.1
Broadcast		1.469	0.308	0.816	5.73	101.0
Average		1.575	0.337	0.845	5.78	69.5
	Control	1.515	0.311	0.843	5.80	136.0
	Superphosphate	1.560 (0.82)	0.347 (0.44)	0.796 (0.57)	5.86	33.2
	Struvite	1.653 (0.49)	0.353 (0.38)	0.895 (0.56)	5.69	39.3
Average		1.576	0.337	0.845	5.78	69.5
Band	Control	1.741	0.368	0.898	5.86	40.0
Band	Superphosphate	1.666 (0.80)	0.362 (0.92)	0.820 (0.61)	5.92	34.9
Band	Struvite	1.640 (0.73)	0.368 (1.00)	**0.902 (0.98)**	5.73	36.4
Broadcast	Control	1.289	0.255	0.789	5.74	232
Broadcast	Superphosphate	1.454 (0.50)	0.332 (0.21)	0.772 (0.87)	5.81	31.5
Broadcast	Struvite	1.666 (0.20)	0.337 (0.20)	0.0888 (0.41)	5.65	42.3
Average		1.576	0.337	0. 845	5.78	69.5

Significant values are in bold.

**Figure 1:**
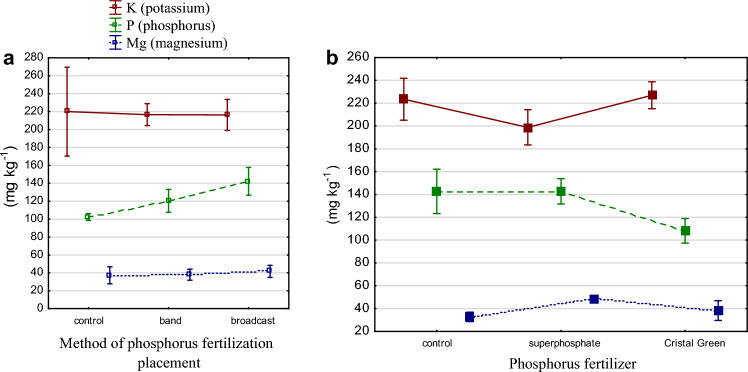
(**a**) Mg, K and P content determined by Egner–Riehm under different fertilizer placement fertilization methods (mg kg^−1^ d m). (**b**) Mg, K and P content determined by Egner–Riehm under phosphorus fertilization (mg kg^−1^ d m).

The method of placement of phosphate fertilizers gave comparable values for K, P and Mg content in the soil (Table 3, Fig. 1a). Phosphorus fertilizer caused differences in the contents of all elements tested. With struvite fertilization, potassium content increased by 2% compared to the control and by 14% compared to triple superphosphate. Magnesium content also increased after struvite fertilization: by 17% compared to the control. Phosphorus content decreased after struvite fertilization; however, this could have been caused by the slightly acidic pH of the soil. Polish soils have a very acidic or acidic reaction and thus affect phosphorus availability. The interaction between factors resulted in an increase in K content where struvite granules were applied near the germinating seeds. The greatest amount of magnesium in the soil was noted under both broadcast and band superphosphate fertilization (Table 3, Fig. 1a,b). The content of phosphorus and potassium in the samples compared to the control was statistically significantly lower (except for K after struvite administration) after administration of each fertilizer using the broadcast method. Mg content, on the other hand, was statistically significantly higher after fertilizer administration, in almost all combinations, than in the control samples.

**Table 3:** Content of available forms of K, P and Mg (mg kg^−1^) determined by Egner–Riehm method (p-value for the t-test with control group).

Method of fertilizer placement	Phosphorus fertilizer	K	P	Mg
Band		217	120	38.0
Broadcast		216	142	41.8
Average		**216**	**131**	**39.9**
	Control	223	142	32.8
	Superphosphate	198 (0.02*)	142 (1.00)	48.5 (0.00*)
	Struvite	227 (0.69)	108 (0.00*)	38.3 (0.17)
Average		**216**	**131**	**39.9**
Band	Control	211	126	35.3
Band	Superphosphate	207 (0.76)	134 (0.27)	47.7 (0.01*)
Band	Struvite	231 (0.16)	100 (0.00*)	31.0 (0.20)
Broadcast	Control	236	159	30.3
Broadcast	Superphosphate	190 (0.01*)	151 (0.07*)	49.3 (0.00*)
Broadcast	Struvite	222 (0.17)	116 (0.00*)	45.7 (0.00*)
Average		**216**	**131**	**39.9**

*Statistically significant α = 0.05.

**Figure 2:**
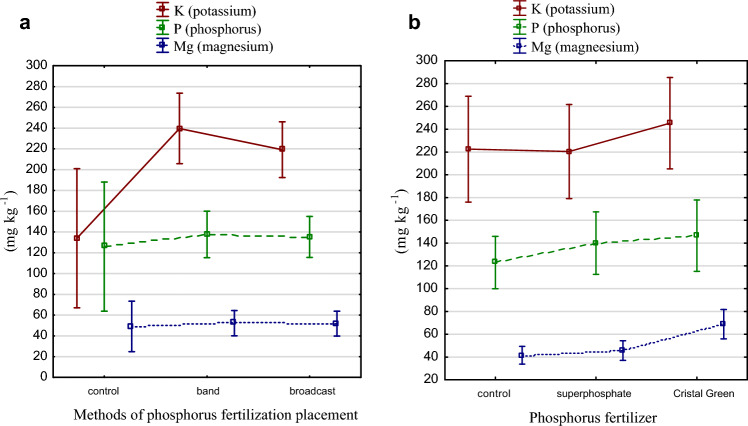
(**a**) Mg, K and P content determined by Mehlich 3 under different fertilizer placement fertilization methods (mg kg^−1^ d m). (**b**) Mg, K and P content determined by Mehlich 3 under phosphorus fertilization (mg kg^−1^ d m).

The method of phosphorus placement differentiated K content in the soil, while P and Mg was at the same level (Fig. 2a). In the Mehlich 3 method, the values for all elements were higher under struvite fertilization. Magnesium content under struvite fertilization increased by 65% compared to the control and 50% compared to superphosphate (Table 4, Fig. 2b). Based on the limit numbers concerning Mehlich 3 developed by Kęsik^22,26^ the phosphorus content of the experiment after the application of superphosphate and struvite taking the soil reaction (5.6–5.8) was determined as average, potassium content—high and magnesium—low. Magnesium content under struvite fertilization increased statistically significantly: by 65% compared to the control and 50% compared to superphosphate (Table 4, Fig. 2b).

**Table 4:** Content of available forms of K, P and Mg (mg kg^−1^) determined by Mehlich 3 method (*p*-value for the *t*-test with control group).

Method of fertilizer placement	Phosphorus fertilizer	K	P	Mg
Band		239	137	52.3
Broadcast		219	135	51.7
Average		**229**	**136**	**52.0**
	Control	222	123	41.5
	Superphosphate	220 (0.94)	140 (0.25)	45.7(0.38)
	Struvite	245 (0.36)	146 (0.15)	68.8 (0.00*)
Average		**229**	**136**	**52.0**
Band	Control	240	124	41.7
Band	Superphosphate	222 (0.66)	133 (0.69)	45.9 (0.59)
Band	Struvite	257 (0.70)	156 (0.27)	69.2 (0.04*)
Broadcast	Control	205	122	41.4
Broadcast	Superphosphate	219 (0.70)	1470 (0.31)	45.5 (0.60)
Broadcast	Struvite	233 (0.36)	137 (0.51)	68.4 (0.04*)
Average		**229**	**136**	**52.0**

*Statistically significant α = 0.05.

**Figure 3:**
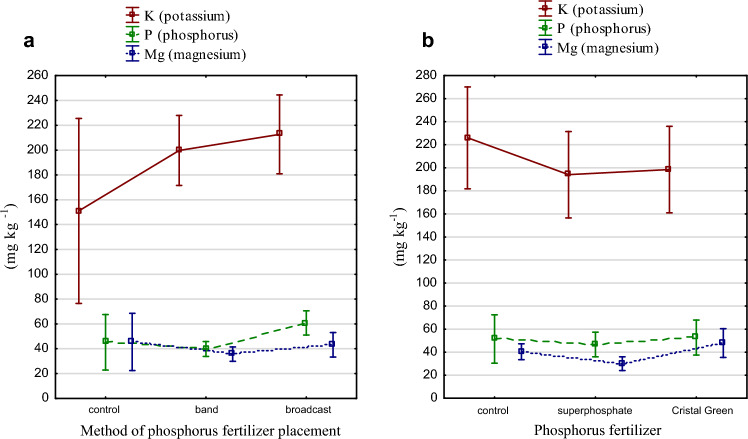
(**a**) Mg, K and P content determined by Yanai under different fertilizer placement phosphorus fertilization methods (mg kg^−1^ d m). (**b**) Mg, K and P content determined by Yanai under phosphorus fertilization (mg kg^−1^ d m).

The greatest content of K, P, Mg was observed under broadcast fertilization (Table 5, Fig. 3a). Struvite fertilization caused increase of P and Mg content in the soil. Again as in the Mehlich 3 method, Mg content increased under struvite fertilization by 19% compared to control and by 60% compared to superphosphate (Fig. 3b). Mg content was statistically significantly lower under superphosphate fertilization by 24% compared to control, with band fertilizer lower by 24% with broadcast by 27%.

**Table 5:** Content of available forms of K, P and Mg (mg kg^−1^) determined by Yanai method (p-value for the t-test with control group).

Method of fertilizer placement	Phosphorus fertilizer	K	P	Mg
Band		199	39.8	35.6
Broadcast		212	60.7	43.2
Average		**206**	**50.3**	**39.4**
	Control	226	51.45	40.3
	Superphosphate	194 (0.19)	46.6 (0.61)	30.1 (0.02*)
	Struvite	198 (0.25)	52.7 (0.90)	47.9 (0.20)
Average		**206**	**50.3**	**39.4**
Band	Control	215 (0.66)	35.4 (0.32)	37.7 (0.63)
Band	Superphosphate	185 (0.40)	41.4 (0.40)	28.7 (0.08)
Band	Struvite	199	42.7	40.5
Broadcast	Control	237	67.5	42.9
Broadcast	Superphosphate	203 (0.40)	51.9 (0.19)	31.4 (0.14)
Broadcast	Struvite	198 (0.33)	62.7 (0.67)	55.3 (0.20)
Average		**206**	**50.3**	**39.4**

*Statistically significant α = 0.05.

### Analysis of correlations between the analytical methods in determining elements in the soil

The Mehlich and Yanai methods give correlated results which means that there is a statistically significant, linear relationship between them (Tables 6, 7). Thus, the indications generated by the methods can be considered proportional. On the basis of statistical analyses, a positive correlation was found between Mg and K content in soil as determined by the Mehlich 3 and Yanai methods. No such relationship was found for the Egner–Riehm method. With regard to phosphorus content, there was no correlation between any method, which may suggest that phosphorus is a sensitive element to determine and depends on many factors, the most important of which is soil pH.

**Table 6:** Relationships between Mg content in soil determined by different methods.

Mg content by different methods	Average	Standard error	Mg by Egner–Riehm	Mg by Mehlich 3	Mg by Yanai
Mg by Egner–Riehm	39.5	7.87	1.000	0.005	− 0.166
Mg by Mehlich 3	51.5	14.44	0.005	1.000	0.695*
Mg by Yanai	40.3	10.75	− 0.166	0.695*	1.000

(N = 21), *statistically significant with α = 0.05.

**Table 7:** Relationships between K content in soil determined by different methods.

K content by different methods	Average	Standard error	K by Egner–Riehm	K by Mehlich 3	K by Yanai
K by Egner–Riehm	216	18.6	1.000	− 0.012	0.078
K by Mehlich 3	215	51.0	− 0.012	1.000	0.789*
K by Yanai	198	41.7	0.078	0.789*	1.000

(N = 21), *statistically significant with α = 0.05.

Phosphorus measurements by each method indicated different results. It is not possible to indicate a linear relationship for the obtained concentrations for any pair of measurement methods with a statistical significance at the α = 0.05 level (Table 8).

**Table 8:** Relationships between P content in soil determined by different methods.

P content by different methods	Average	Standard error	P by Egner–Riehm	P by Mehlich 3	P by Yanai
P by Egner–Riehm	127.2	22.1	1.000	− 0.115	0.395
P by Mehlich 3	135.0	26.1	− 0.115	1.000	0.143
P by Yanai	49.5	14.1	0.394	0.143	1.000

(N = 21).

**Figure 4:**
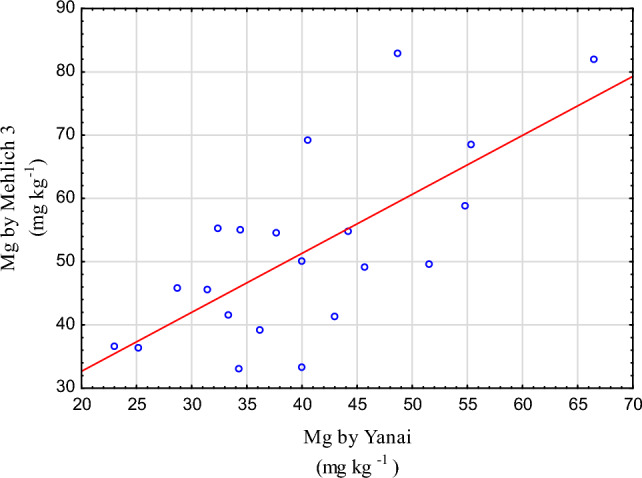
Scatter of Mg content in soil determined by the Yanai and Mehlich 3 methods.

The statistical significance of the linear correlation is confirmed by the scatterplot in Fig. 4. The regression line $Mg\left(M\right)=0.932\times Mg\left(Y\right)+14.0$ with a statistically significant (with ∝  = 0.05) slope of 0.932 fitted with the least squares method allows an estimation of the results for one method based on the values obtained with another method. All linear regression assumptions are met (the model residuals conform to the normal distribution, residuals heteroscedasticity, no autocorrelation of residuals). The estimation is subject to a large error (standard estimation error 10.6) and goodness-of-fit measure ${R}^{2}=0.48$.

### Analysis of variance of tested element content under different phosphorus placement and phosphorus fertilizer

A significant effect of phosphorus fertilization on soil K content was found by determining this element using the Egner–Riehm method. No such relationship was found using the other two methods (Table 9).

**Table 9:** Two-factor analysis for K content under different phosphorus placement and phosphorus fertilizers.

Potassium	Egner–Riehm method	Mehlich 3	Yanai
Method of phosphorus fertilizer placement (A)	0.969	0.333	0.516
Phosphorus fertilizer (B)	0.003*	0.556	0.377

*Significant difference α = 0.05.

The method of phosphorus fertilizer placement had a significant effect on the phosphorus content of the soil as determined by the Egner–Riehm and Yanai methods. Phosphorus fertilization significantly modified the content of this element in the soil as determined by the Egner–Riehm method (Table 10).

**Table 10:** Two-factor analysis for P content under different phosphorus placement and phosphorus fertilizers.

Phosphorus	Egner–Riehm method	Mehlich 3	Yanai
Method of phosphorus fertilizer placement (A)	0.000*	0.863	0.001*
Phosphorus fertilizer (B)	0.000*	0.357	0.577

*Significant difference α = 0.05.

The method of phosphorus fertilizer placement had a significant effect on the soil magnesium content determined by the Egner–Riehm method. Phosphorus fertilization significantly affected the content of this element as determined by the three methods being tested (Table 11).

**Table 11:** Two-factor analysis for Mg content under different phosphorus placement and phosphorus fertilizers.

Magnesium	Egner–Riehm method	Mehlich 3	Yanai
Method of phosphorus fertilizer placement (A)	0.007*	0.922	0.057
Phosphorus fertilizer (B)	0.000*	0.001*	0.005*

*Significant difference α = 0.05.

## Discussion

In this study, we evaluated the performance of three methods in P, Mg and K determination in soil under struvite fertilization in soybean cultivation. Sims et al.^27^ claimed that the application of phosphorus fertilization in the form of organic waste and phosphorus fertilizers on the basis of soil abundance requires a test that is integrated with the requirements of agricultural practices for the management of this nutrient and that takes into account environmental risks.

In our study, P content under struvite decreased after soybean harvest, unlike in the study by Bastid et al.^28^ where P availability was highest in soil with struvite. In the study by Bastid et al.^28^, P availability was lower after one month. This decrease in P availability over one month may be due to P precipitation as Al, Fe and Ca phosphates and uptake by plants^29,30^. This decrease was also observed in our study and can also be explained by P precipitation, which is indicated by low pH. pH and salinity are the main factors influencing P sorption–desorption behaviours in soils^31^. Liu et al.^32^ observed that P sorption on sediments increases at low salinity, while it decreases as salinity increases (> 5‰). Struvite fertilization causes a decrease in salinity. Our study indicates a P sorption increase. In our experiment, the application of struvite reduced the phosphorus content in the soil. Under the influence of the application of struvite, the soil reaction decreased further. Phosphorus fertilization can cause a not dissimilar increase in the content of the available forms of phosphorus in the soil. In acidic and slightly acidic soils, phosphorus in the forms of leachable and P–Al can occur. This needs further research to determine the different forms (including fractions: easily soluble, exchangeable, bound to organic matter, bound to stable organic-mineral and mineral compounds, residual) of P. The available contents of P, K, Ca and Mg can decrease over time after fertilization as a result of their absorption and uptake by plants.

Soil fertilized with struvite presents slightly lower available P content and higher available Mg content, and this may be connected to the higher solubility of superphosphate according to Ref.^33^ and the higher amount of Mg contained in struvite.

In our study, struvite fertilization did not have a more significant effect on seed yield and phosphorus uptake than that seen in other studies^15,34,35^ but it did have an effect on seed phosphorus content. Despite the lack of significant differences, greater values of seed yield and P uptake can be attributed mainly to the greater amount of Mg contained in *Crystal Green* fertilizer and its synergistic effect on P uptake^36^. In agreement with^34^ P and Mg uptake were significantly dependent on P fertilization, while P dose had no significant effect on lettuce.

Phosphorus fertilization requires efficient fertilization technologies; thus, this issue also concerns struvite. In Talboys et al.^9^, struvite granules placed with the seed did not provide the same level of phosphorus supply as the DAP (diamonium phosphate) granules for early-growing spring wheat. However, it gave equivalent rates of P uptake, yield and apparent fertilizer recovery at harvest, even though only 26% of the struvite granules had completely dissolved. In our study, phosphorus content was higher in the soil placed broadcast. P diffusion when placed band to the root system is high because of the large concentration gradient compared with broadcast P placement. However, when bulk soil test P is high, the benefits of banded P are reduced, and this was probably the case in our study. Therefore, knowledge of soil test P levels is pivotal when determining placement options. The optimal balance between P banding and broadcast application is difficult to achieve^37,38^. Roots take up phosphate in the form of H_2_PO_4_ ions. When the pH is lowered from 6 to 4, the rate of phosphate uptake by roots increases, the amount of phosphate desorbed from the soil increases, and the amount of phosphate sorbed by the soil often increases, but this is not confirmed in our study, where surface-applied phosphorus supplied the soil with this element.

Fei et al.^39^ showed that long-term, continuous implementation of phosphorus into greenhouse soil—both in the form of mineral and natural (manure) fertilizers—used in vegetable production, caused its accumulation in the soil and enrichment of its top layers. The authors found a fourfold increase in total phosphorus content after 13 years of its use compared to the initial value. The total phosphorus content of soil usually varies between 500 and 800 (50–3000) mg P kg^−1^ soil and depends on many factors. The highest amount is found in the near-surface layers of the soil and decreases with the depth of the soil profile. In our study, the total P content varies from 255 to 366 mg kg^−1^ d m.

Our research shows that struvite is a good source of magnesium. Compared to the control, a noteworthy increase in soil magnesium content under the application of struvite is determined by the Yanai, Mehlich 3 and Egner–Riehm methods. STR is a source of P needed by the fertilizer market with agronomic and environmental benefits such as providing available Mg. However, the specific mechanism of Mg-release from soil after struvite application remains unexplained. In Poland, sandy soils cover several million hectares. Most are, simultaneously, under-fertilized and magnesium-deficient soils. Thus, further research should be conducted. Fertilizer application could solve the magnesium deficit occurring in the soils of Central and Eastern Europe. As in our study, a significant increased content of magnesium was also found by Szymanska et al.^13,40^ in the first year after the application of struvite.

According to the Egner–Riehm method, the application of struvite resulted in a significant increase in soil potassium content. In the study by Kas et al.^41^, organic fertilization increased the K content of soils almost two-fold compared to mineral fertilization. Significantly lower K content was found in soils with control and NPK. Cong et al.^42^ showed that deficiencies in N, P and Mg reduced the effectiveness of applied K and may have been the cause of increased leaching of K from the arable layer; this is consistent with our study. Struvite contains N and P in its composition and, compared to superphosphate used on this soil, K content increased. This increase may affect the concentration of K in the soil due to the antagonism that can appear between K and Mg^42^.

Chemically, phosphorus is a low-mobility element available to plants only direct from the space of the roots and its uptake is highly dependent on soil reaction and temperature; hence, the idea of the above study^43^. The forms in which phosphorus is accumulated in the soil depend on management practices, fertilizer sources and application methods, which can promote different solubility of phosphorus and its uptake by plants^44^.

The Mehlich and Yanai methods allows examination in a single soil extract of not only the content of basic macronutrients such as phosphorus, potassium and magnesium, but also other important nutrients, e.g. micronutrients such as boron, copper and others^24^. The result is a significant reduction in energy and water consumption, as well as a marked reduction in labour intensity and reagent costs. This makes the method cheaper and more environmentally friendly. From the farmer’s point of view, the most important advantage of the Mehlich 3 method is the cost of determining P, K and available Mg (and pH) in one soil sample. The cost of element determination with the Egner–Riehm method is around 27% higher compared to Mehlich 3. Mehlich 3 method has been gradually implemented in Poland since 2015. Limit numbers have been determined for each element for Mehlich, while no such numbers have been determined for the Yanai method resulting in a lack of interest in introducing this method on a larger scale^22,25,26^.

## Materials and methods

### Experiment design

A greenhouse experiment was established to assess the effect of struvite fertilization on content elements in soil under soybean cultivation. The 0–30 cm soil depth was used in the experiment with samples from the Experimental Station of Wroclaw University of Environmental and Life Sciences (Pawłowice) (geographical location 17° 7′ E and 51° 08′ N in the Lower Silesian Voivodship, Wrocław, Poland). A pot experiment was established in 2022 at the Experimental Station of Wroclaw University of Environmental and Life Sciences (Pawłowice) using fertilizer produced from sewage sludge (*Crystal Green*) in soybean cultivation. The two-factor experiment was conducted in six replications. The first factor was the differential placement of phosphorus fertilizer (band and broadcast). Surface fertilization consisted of random placement of fertilizers on the surface of the pot, while broadcast was done by placing fertilizer granules at a depth of about 5 cm below the sown soybean seed. The second factor was differentiated phosphorus fertilizers against the control. Two phosphorus fertilizers were used in the experiment: traditional triple superphosphate (SUP), commonly used in soybean cultivation, and *Crystal Green* (CG). In the experiment, the effect of a fertilizer produced on the base of sewage sludge with the trade name *Crystal Green* (produced by Ostara Nutrient Technologies) was studied in comparison with the traditional fertilizer, triple superphosphate^26^. The white granules of struvite measured around approximately 1–2 mm in diameter. Phosphorus recovery comprised phosphorus minerals precipitation from sewage sludge as struvite (magnesium ammonium phosphate hexahydrate, MgNH_4_PO_4_ 6H_2_O). Struvite contains N (2%), P (24%) and Mg (10%) and is characterized by a low heavy metal content compared to triple superphosphate^7,15^. From the chemical point of view, it is not pure struvite.

The total number of pots was 36. The pot diameter was 20 cm, depth 20 cm and the volume around 5000 cm^3^. The pots were filled with soil which was mixed with SUP, CG, nitrogen and potassium fertilizer. The particle size distribution of the mineral parts corresponded to sandy clay.

Soil with the following parameters was used in the experiment:

Mehlich 3 method: P—126, K—134, Mg—49 mg kg^−1^ d m (average content of P, according to limit numbers, K—low content, Mg—low content)^26,45^.

Yanai method: P 45.2, K—151, Mg—45.6 mg kg^−1^ d m (limit numbers have not been developed).

Egner–Riehm method: P—103, K—220, Mg—38 mg kg^−1^ d m (P—average content, K—high content, Mg—low/average content)^22,45^.

Fertilizer doses in the experiment were based on the optimum for growing soybeans under field conditions, i.e. 70 kg ha^−1^ P_2_O_5_, 120 kg ha^−1^ K_2_O, and a starter dose of nitrogen 30 kg ha^−1^ N. Only nitrogen and potassium were applied at the same dose. The following fertilizer doses per pot were applied (converted):

Struvite—0.76 g,

Superphosphate—0.54 g,

Ammonium nitrate—0.27 g,

Potassium salt—1.25 g.

Abellina soybean seeds were sown into pots of 4 in the second decade of May 2022. The treated soybean seeds were provided by Saatbau and had been inoculated^46^. Soybean seeds were inoculated within Fix Fertig technology which involves factory-coating seeds with bacteria that are dormant. The seeds were coated with *Bradyrhizobium japonicum* bacteria.

Prior to sowing, germination capacity was determined based on current standards. The germination capacity of the tested variety averaged 75%. The number of seeds sown per pot was based on the optimal density of soybean seeds under these conditions. No significant pests or weeds were found in the soybean during the experiment, so the use of herbicides was not necessary. Soybeans were watered regularly.

### Plant material sampling and chemical analyses

Samples for chemical analysis were taken after the end of the growing season. The content of phosphorus, magnesium and potassium in the plant material was determined colorimetrically: P using ammonium vanadomolybdate, Mg using the titanium yellow method, and K colorimetrically. The uptake of P, Mg and K was based on soybean seed yield and the content of these macronutrients in soybean seeds. Seed yield was converted per hectare at 15% moisture content.

### Soil sampling

Soil samples were taken from the 0–20 cm layer after the end of the soybean growing season (October 2022) using a Egner stick. For broadcast and band fertilization treatments, soil samples from each pot were created by subsampling. Soil from three or four locations was then mixed to form a subsample. This procedure was repeated three times for each pot, and these were mixed to form a surface sample. Soil samples were air-dried, disaggregated using a porcelain pestle and mortar, and sieved to < 2 mm. A portion of each sample was further finely ground for analysis. The soil for the pots was sieved through a sieve with a mesh diameter of 10 mm. The particle size distribution of the mineral part were determined using a Mastersizer 2000 laser diffractometer.

#### Soil chemical analyses

Soil pH was measured with a glass pH electrode (1:5 soil:deionized water, measurements after 30 min) and conductivity was assessed using a conductivity meter (the conductivity method). Total phosphorus, potassium and magnesium content was determined after microwave mineralization while available forms were determined with the Egner–Riehm, Mehlich 3 and Yanai methods.

Phosphorus and potassium compounds in a soil according to the Egner–Riehm method were extracted with a lactate buffer consisting of calcium lactate and lactic acid. The described reaction occurs according to the formula:

$${\text{2CH}}_{{3}} \left( {{\text{CHOHCOO}}} \right)_{{2}} + {\text{ 2HCl }} \leftarrow \, \to {\text{ Ca}}\left( {{\text{CHOHCOO}}} \right)_{{2}} + {\text{ 2CH}}_{{3}} {\text{CHOHCOOH }} + {\text{ CaCl}}_{{2}} .$$

The extraction solution used had pH = 3.55 (at this level of acidity, extraction conditions are maintained regardless of the initial soil reaction)^26^. Phosphorus, potassium and magnesium content in soil was determined according the Egner–Riehm method, see details in Table 12.

**Table 12:** Overview of the studied soil element extraction methods used in the research.

Method	Extracting solution	Solution pH	Soil-to-solution ratio	Extraction time	Concentration	References
Egner–Riehm	Calcium lactate	3.6	1:50	90 min	0.04 mol l^−1^ of calcium lactate	Egner et al.^48^
Mehlich 3	Acetic acid, ammonium nitrate, ammonium fluoride, nitric acid and EDTA	2.5	1:10	5 min	0.2 mol l^−1^ CH_3_COOH, 0.25 mol l^−1^ NH_4_NO_3_, 0.015 mol l^−1^ NH_4_F, 0.013 mol l^−1^ HNO_3_, 0.001 mol l^−1^ EDTA	Mehlich^23^
Yanai	Acetic acid, ammonium chloride, citric acid monohydrate, hydrochloric acid	1.3	1:10	30 min	0.2 mol l^−1^ CH_3_COOH, 0.25 mol l^−1^ NH_4_Cl, 0.005 mol l^−1^ C_6_H_8_O_7_, 0.05 mol l^−1^ HCl	Yanai et al.^25^

All extracts were measured using ICP-OES.

The air-dry soil sample was extracted with Mehlich 3 solution (a solution containing 0.2 mol l^−1^ CH_3_COOH, 0.25 mol l^−1^ NH_4_NO_3_, 0.015 mol l^−1^ NH_4_F, 0.013 mol l^−1^ HNO_3_, and 0.001 mol l^−1^ EDTA). The air-dry soil sample was extracted with Mehlich 3 solution by volume ratio of 1:10. For this purpose, two grams of dry soil was weighed into an extraction vessel (a plastic bottle with a capacity of about 150 ml) and then 20 ml of Mehlich solution 3 was added. The sample was shaken for 5 min at 220 cycles min^−1^ on a shaker machine and filtered through medium filters. The solution prepared in this way was analysed for potassium, phosphorus and magnesium content using inductively coupled plasma emission spectrometry ICP-OES (Table 12).

An air-dry soil sample was extracted with Yanai solution (a solution containing 0.2 mol l^−1^ CH_3_COOH, 0.25 mol l^−1^ NH_4_Cl, 0.005 mol l^−1^ C_6_H_8_O_7_, and 0.05 mol l^−1^ HCl) with a volume ratio of 1:10. For this purpose, 5 g of soil were weighed into a 100 ml plastic extraction bottle, 50 ml of extractant was added and the mixture was shaken for 30 min at a rate of 180 cycles min^−1^. Shaking was done on a soil shaker. The solution was then filtered through Advantec Toyo filter paper No.5. The solution prepared in this way was analysed for potassium, phosphorus and magnesium content using inductively coupled plasma emission spectrometry ICP-OES. A sample of the resulting soil extract was carried out into mist form and transferred to a plasma torch, where it was excited at a high frequency and measurement of atomic emission of radiation for the appropriate wavelengths was performed.

### Statistical analyses

Data from chemical analyses (P, Mg, K, pH, salinity) were subjected to Anova/Manova statistical analysis in Statistica software (version 13.1 StatSoft, Poland)^47^. The level of significance was α = 0.05. One-way and two-way analyses of averages were performed to determine the effects of P fertilizer on chemical analyses of soil. Correlations and figures have been prepared using Statistica software.

## Conclusions

In summary, it should be concluded that traditional phosphorus fertilizers can be substituted with struvite, which would simultaneously support a circular economy. Based on the results of the study, there was an increase of 34–37% in available magnesium in the soil after struvite application compared to superphosphate; this was dependent on the analytical method (Mehlich 3 and Yanai). Available phosphorus content in the soil increased under struvite application by 4% relative to superphosphate and 16% relative to the control (Mehlich 3). However, long-term field experiments are needed as a way to measure sustainable phosphorus fertilization in agriculture, as they contribute to a better understanding of the effects of struvite and examine which fractions of phosphorus will dominate in the soil after struvite application in regard to the care and protection of the soil environment. Additionally, similar field experiments are also required to evaluate the soil macronutrient abundance classes for the Yanai method, which—due to its lower cost compared to the Egner–Riehm method—has the ability to determine a larger pool of elements than the other two methods. Therefore, in the case of the introduction of the Yanai method into analytics at chemical-agricultural stations, it would be necessary to develop limit values based on such long-term field experiments.

## Data Availability

All data generated or analysed during this study are included in this published article. Experimental research and field studies on plants (either cultivated or wild), including the collection of plant material are complying with relevant institutional, national, and international guidelines and legislation. I declare that the plant material used for our study was purchased from SAATBAU company in Środa Śląska (Poland) as seed material. We did not use endangered plant species for the experiments.
